# Does repeating a grade affect later life executive functioning? Findings from the Health and Retirement Study

**DOI:** 10.1093/geroni/igaf122.2455

**Published:** 2025-12-31

**Authors:** Rachel Missell-Gray, Ya-Han Chang, Silvia Sörensen

**Affiliations:** University of Rochester, Rochester, New York, United States; University of Rochester, Rochester, New York, United States; University of Rochester Warner School of Education and Human Development, Rochester, New York, United States

## Abstract

Two-thirds of American adults report experiencing at least one Adverse Childhood Experience (ACE).1 ACEs demonstrate negative effects on executive functioning (EF), and EF difficulties in young children are associated with continued academic difficulties.2 In past decades, this meant repeating a grade. Studies suggest ACEs may affect adult cognition3, yet the mechanisms of this association remain underexplained. One mechanism may be that repeating a grade as a result or cause of early life stress, potentially affects later life EF. We investigated whether older adults who repeated a year of school performed poorly on Trails B, an EF task of rule switching and working memory. We examined 2,657 community-dwelling older adults from the Health and Retirement Study (HRS) with data from the 2016 Life History Survey and the Harmonized Cognitive Assessment Protocol. All respondents had education and Trails B data. Study outcomes were Trails B scores, and the primary independent variable was repeating a year of school, controlling for age, degree, and race/ethnicity. Repeating a year of school was associated with lower Trails B scores (β = -.064, p <.001). Linear regression models found that final degree and repeating a year of school accounted for 13% of the variance in Trails B scores (R2 =.13, F(1, 2649)=11.94, p <.001). Together, age, race/ethnicity, final degree, and repeating a year of school explained 25% of the variance in Trails B scores (R2 =.25, F(2, 2644)=75.94, p <.001). Future research could examine possible mechanisms (e.g., decreased opportunity) by which education may impact later life EF.

